# Midwives’ experiences of implementing respectful maternity care knowledge in daily maternity care practices after participating in a four-day RMC training

**DOI:** 10.1186/s12912-021-00559-6

**Published:** 2021-03-10

**Authors:** Veronica Millicent Dzomeku, Adwoa Bemah Boamah Mensah, Emmanuel Kweku Nakua, Pascal Agbadi, Jody R. Lori, Peter Donkor

**Affiliations:** 1 Department of Nursing, Faculty of Allied Health Sciences, College of Health Sciences, Kwame Nkrumah University of Science and Technology, Kumasi, Ghana; 2 Department of Epidemiology and Biostatistics, School of Public Health, Kwame Nkrumah University of Science and Technology, Kumasi, Ghana; 3 School of Nursing, University of Michigan, Ann Arbor, USA; 4 Department of Surgery, School of Medical Sciences, Kwame Nkrumah University of Science and Technology, Kumasi, Ghana; 5 Komfo Anokye Teaching Hospital, Kumasi, Ghana

**Keywords:** Respectful maternity care training, Obstetric violence, Midwives, Ghana

## Abstract

**Background:**

In Ghana, studies documenting the effectiveness of evidence-based specialized training programs to promote respectful maternity care (RMC) practices in healthcare facilities are few. Thus, we designed a four-day RMC training workshop and piloted it with selected midwives of a tertiary healthcare facility in Kumasi, Ghana. The present paper evaluated the impact of the training by exploring midwives’ experiences of implementing RMC knowledge in their daily maternity care practices 4 months after the training workshop.

**Methods:**

Through a descriptive qualitative research design, we followed-up and conducted 14 in-depth interviews with participants of the RMC training, exploring their experiences of applying the acquired RMC knowledge in their daily maternity care practices. Data were managed and analysed using NVivo 12. Codes were collapsed into subthemes and assigned to three major predetermined themes.

**Results:**

The findings have been broadly categorized into three themes: experiences of practising RMC in daily maternity care, health facility barriers to practising RMC, and recommendations for improving RMC practices. The midwives mentioned that applying the newly acquired RMC knowledge has positively improved their relationship with childbearing women, assisted them to effectively communicate with the women, and position them to recognize the autonomy of childbearing women. Despite the positive influence of the training on clinical practice, the midwives said the policy and the built environment in the hospital does not support the exploration of alternative birthing positions. Also, the hospital lacked the required logistics to ensure privacy for multiple childbearing women in the open labour ward. The midwives recommended that logistics for alternative birthing positions and privacy in the ward should be provided. Also, all midwives and staff of the hospital should be taken through the RMC training program to encourage good practice.

**Conclusion:**

Despite the report of some RMC implementation challenges, the midwives noted that the 4-day RMC training has had a positive impact on their maternity caregiving practice in the hospital. Policies and programs aimed at addressing the issue of disrespect and abusive practices during maternity care should advocate and include the building of facilities that support alternative birthing positions and privacy of childbearing women during childbirth.

**Supplementary Information:**

The online version contains supplementary material available at 10.1186/s12912-021-00559-6.

## Background

The concerted efforts by international and local organizations to reduce maternal mortality is motivated by the belief that “no woman should die while giving life”. The reduction in obstetric related deaths in the past three decades is worth celebrating [1]. However, the death of many women during childbirth in many lower-and-middle-income countries (LMICs) call for drastic life-saving measures that promote safe pregnancy and childbirth. Several policies, programs, and interventions were pursued to end obstetric related deaths in sub-Saharan African countries and other developing countries [2, 3]. These countries vigorously embarked on programs that encouraged childbearing women to deliver in healthcare facilities [4, 5]. Despite its positive impact, facility-based deliveries have become a source of worry to many women due to frequent reports and experiences of obstetric violence and abuse in the hands of maternity care providers [1, 6]. These type of violence and abuses are partly responsible for the death and disability of many women during pregnancy and childbirth [1].

Research shows that obstetric violence and abuse is common in many healthcare facilities in sub-Saharan Africa. These abuses have led many women’s and children’s health researchers, advocates, and policymakers to begin several initiatives to promote respectful and non-abusive maternity care during pregnancy, childbirth, and in the time after birth. The truss of these respectful maternity care efforts is the deep recognition that childbearing women have inalienable rights to quality care and a zero-tolerance for violence and abuse [7, 8]. Respectful maternity care (RMC) is defined as the “care organized for and provided to all women in a manner that maintains their dignity, privacy and confidentiality, ensures freedom from harm and mistreatment, and enables informed choice and continuous support during labour and childbirth” [8]. Dignified care is the childbearing woman’s right to be valued as human and treated ethically.

A growing body of evidence shows that the implementation of specialized respectful maternity care programs and interventions can effectively change the way midwives administer care in developing countries to reduce mistreatment during intrapartum care services [9–15]. Some of these programs and interventions include theatre-based training interventions to reduce obstetric violence [14], monitoring of midwives during practice [9], improvement in working conditions of healthcare providers [11], equipping midwives with the ability to identify and manage obstetric and neonatal emergencies [16], and the training of midwives to effectively communicate with clients [10, 17].

In Ghana, studies documenting the effectiveness of evidence-based specialized training programs and interventions to promote respectful maternity care practices in healthcare facilities are dearth [16]. Thus, we designed a four-day respectful maternity care training workshop and piloted it with selected midwives of a tertiary healthcare facility in Kumasi, Ghana. The training modules equipped the trainees in the best practices that promote quality intrapartum care devoid of violence and abuse through the implementation of effective, alternative birthing positions, focused antenatal care, empathetic and ethical communication with childbearing women, and demonstrating respect and dignity during intrapartum care provision. The present paper aims to evaluate the impact of the four-day training by exploring midwives’ experiences of applying RMC knowledge in their daily maternity care practices 4 months after the training workshop.

## Methods

### Design

We adopted a qualitative descriptive design. This research design was suitable because of our study objective. Our choice of this method further echoed our need to simply describe how trained midwives are providing intrapartum care in a respectful and dignified manner after having participated in a training that equips them with special knowledge and skills in the provision of respectful maternity care. Qualitative descriptive design is useful in situations where researchers are interested in obtaining broad insights and rich information about a phenomenon to develop and refine interventions [18].

### Study setting

Our study is situated in a tertiary health facility in Kumasi, located in the Ashanti region of Ghana. This facility provides healthcare services to patients across the country and has a bed capacity of about 1200 and a staff strength of about 3000. It is the main referral hospital for the Ashanti, Brong Ahafo, Western, the three northern regions (Northern, Upper East, And Upper West), and neighbouring countries. It has thirteen (13) clinical directorates (departments) one of which is the Obstetrics and Gynaecology (O &G) directorate, which has four labour wards. In 2018, the hospital recorded an estimated 4792 Spontaneous vaginal deliveries, an estimated 123 maternal deaths, and 61 neonatal deaths [KATH O & G Records, 2019]. In 2017, an estimated 308 women per 100, 000 live births have died [19].

### Participant recruitment and data collection

Fifteen midwives who participated in our four-day Respectful Maternity Care-Module (RMC-M) training program were approached for this study. Fourteen of them responded and consented to be interviewed. Thus, we conducted 14 in-depth interviews with the recruited participants. One participant was on study leave at the time of the study. The interviews were guided by a semi-structured protocol in line with the study’s objective (Additional File 1). We conducted the interviews between June and July 2019. ABBM, the second author, conducted the interviews. She has vast clinical and academic experiences in qualitative research, nursing practice, women’s health, with specializations in women health and cancers. Also, she is fluent in both “Twi” and the English language, which were both acceptable mediums for conducting the interviews because they were the predominant languages spoken in the study setting. The interviews were conducted inside an office in the study setting at the request of the participants to make it flexible and convenient for them because of their work. Each interview lasted approximately an hour, and with the written consent of the participants, the session was audio-recorded and transcribed.

### Brief description of respectful maternity care-modules and training

A training manual, called Respectful Maternity Care Modules (RMC-M), was used to guide the four-days training intervention for fifteen midwives purposively selected at the study site. The RMC-M comprises of four modules. The manual was developed from the data collected during the first author’s PhD project on disrespect and abuse in intrapartum care at four public hospitals in Ghana and from the literature on the state of the science on maternity care [20]**.** The manual contains detailed contents on each module: 1) respect and dignity in childbirth; 2) communication; 3) focused antenatal care; and 4) use of alternative birthing positions for delivery. The training was facilitated by the first and second authors and two midwifery educators in Ghana. It employed interactive teaching and learning methods such as role-play, discussion, brainstorming, demonstration, and case study to inculcate in midwives acceptable practices, skills, and attitudes for respectful care provision.

### Module one: respect and dignity in childbirth

This module equips midwives on childbearing women’s rights, respectful childbearing women care, ethics and ethical issues in nursing/midwifery practise, and practical issues in childbearing women care.

### Module two: communication during childbearing women care

This module equips midwives on communication, forms of communication, barriers to effective communication, techniques for active listening, and seeking consent in maternity care.

### Module three: focused antenatal care (FANC)

This module helps midwives to understand the principles of FANC, differentiate between FANC and traditional antenatal care, basic and specialised components of antenatal care, schedule, objectives and procedures in FANC, and birth preparedness and complication readiness plan in maternity care.

### Module four: effective, alternative birthing positions

This module explains the main cost-effective positions [birthing bar, birthing stool, sitting upright, kneeling, semi-seated, with support, and side, curled position] used in childbirth, the advantages of the positions, the skills required to teach childbearing women about the positions, and provide a practical demonstration on the use of these positions during childbirth.

### Data management and analysis

The second author transcribed the recorded interviews verbatim. The third (NKE) and fourth (AP) authors proofread the transcribed interviews alongside listening to the audio files to ensure that participants’ views were accurately captured. NKE and AP have academic and research expertise in biostatistics, public health, and research methods. Two independent translators fluent in both the Twi and English languages then translated the 14 anonymized “Twi” transcripts using the process of back-back translation while maintaining confidentiality. The first and fourth authors analyzed the data independently. DMV is equally an experienced nurse-midwife and academic in the field of general women’s health with specialization in respectful maternity. The transcripts for all the 14 participants were independently read repeatedly by the first author (DMV) and the fourth author (AP) to make sense of the participants’ experiences of implementing respectful maternity care knowledge in their daily maternity care practices. Data were managed and analysed in NVivo-12 [21]. Insights from the transcripts were broadly presented in line with the main questions in the semi-structured guide. The findings were validated by the first, second, and third authors and through member checking as well.

Data analysis followed the following steps. Three major themes were predetermined from the semi-structured questionnaire. The themes are the implementation of RMC in daily clinical practice, health facility barriers to practising RMC, and midwives’ recommendations for improving RMC practices. These themes were entered into the NVivo software as the main nodes to structure the data analysis. We generated codes and collapsed them into subthemes. These subthemes were assigned to the predetermined major themes. Clear quotes are selected and presented under the subthemes in the results section.

### Rigour and trustworthiness

We are aware of the principles of confirmability, transferability, and authenticity as cardinal strategies in qualitative research. Thus, we achieved them through the following activities. The results, as already mentioned, was reviewed and validated by the one who conducted the interviews, the project lead, and NEK to authenticate the results. Also, to ensure that we are not misrepresenting the views of participants, we have discussed the results following our analysis with four of the participants so they can judge whether we have understood them and accurately presented their views. To aid potential transferability and replicability, we have adequately described our research objective, the research design, data collection method, analysis, as well as provided information on the study setting and the participants.

### Demographic characteristics of participants

The midwives were on average 33 years old, with a range of 31–48 years. They had engaged in professional practice for an average of 8 years.

## Results

This section is organized around themes that were derived from the main research objective. The main themes structuring the findings are the implementation of RMC in daily clinical practice, health facility barriers to practising RMC, and recommendations for improving RMC practices.

### Implementation of RMC in daily intrapartum care practices

We explored midwives’ experiences of their daily implementation of RMC in daily intrapartum care. Midwives cited examples of the RMC caregiving practices they have implemented as a result of their participation in the RMC training program. Also, they indicated the changes they have observed in how they related to childbearing women who were in the healthcare facility for intrapartum care services. We broadly categorized their perceived improvement in their caregiving practices under these subthemes: perceived positive midwife-patient relationship, communicating effectively with childbearing women, and recognizing the autonomy of childbearing women.

#### Perceived positive midwife-patient relationship

The midwives indicated that their relationship with childbearing women before the training was not cordial. According to them, they paid little attention to the views of their clients. However, their relationship with the childbearing women changed after participating in the RMC training. Some of the things they do now that matter to their clients are addressing the women by their names. Some of them indicated that they no longer use abusive and provocative words in addressing their clients regardless of the provocative treatment they received from them. The quotes below are a synopsis of midwives’ accounts of the impact of the RMC training on their practice.

*After getting the knowledge, I realized most of the patients, when they come, already have the misconception ‘nurses are like this, are like that’. But because I had that at the back of my mind, anytime they come, even if they misbehave I talk nicely to them, at the end of the day, we end up creating a very good and cordial relationship.* Midwife 006.

*The training has helped me in managing my clients very well. Now, I do respect their views. Then, any questions they ask, I give them answers. Not like how I used to be before the workshop. I do give them answers and then a hearing ear to their concerns. So it has changed me a lot...Now, I address each patient by her name. Also, I do provide privacy when providing for them, and I don’t use abusive words at them...There were things I was doing before the workshop, now I don’t do (them) anymore. Now, I am cool and calm with the patient and if a patient is trying to provoke me, I will just keep quiet and walk away. Not like at first if you provoke me, I might also say something to you, but now, it has changed. I wouldn’t do that anymore.* Midwife 009.

#### Communicating effectively with childbearing women

The RMC training has had a positive impact on how midwives communicated and treated the childbearing women in the hospital. According to the midwives, clients usually come to the facility with a preconceived notion that they will be mistreated. This notion can sometimes affect the relationship between the midwives and the clients during caregiving. Before the training, the midwives mentioned that they were less concerned about the misconceptions of the childbearing women. After the training, however, they decided to communicate with the women to change their misconceptions about caregiving practices in the facility; they reassured the women and help them to allay their fears. The midwives share the following as their experiences with the childbearing women.

*Because, like at first, they [the childbearing women] just come, I am only doing my nursing skills, I don’t care about what, at times the patient will go like, the baby is at MBU, so when they come and you ask them ‘how is the baby doing?’ And you want to know more but at first, I will, well, your baby is there and you’ve gone to look for the baby. So I don’t care. You just come, you greet and you go back to your cubicle. I wouldn’t ask further ‘how is the baby’, I wouldn’t; I’ll just sit down. But right now, when they come, I ask them ‘how is the baby?’ and if there is anything, I just reassure them but at first I wasn’t doing that unless maybe the patient opens up.* Midwife 003.

*Before then [the training], [when] the patient comes, I just pick the A&C card [Antenatal Card), [and say], ‘Maame, what brought you here?’ Then, she [the pregnant woman] talks. But now [after the training], the moment I see her coming, I smile, because I believe I am rendering service to her, and if she is not there, I will also not be there. And I owe her service, so I have to be nice to her, so when she comes, I smile towards her ‘Oh Maame, how have you been? Why have you been referred here?’ … those kind words … And when they come and maybe the place is full, sometimes I even get up and give my seat to them. But at first, to be frank, I was not doing that (laughing). Midwife 006.*

*Right now, we are trying to impact what we learnt at the workshop at the workplace, so we are doing our best to do that. Sometimes too, because you are trying to do that, the patient can open up to us, and we are happy after their deliveries; they are free with us because we tried to respect their concerns and all that. So that’s what we’ve been doing...when they come for admission, right from the start, we start the respectful care. We sometimes, ask about their concerns, we try to allay their fears and we address their fears, maybe their misconceptions about this place; some people have some tag about the staff in this hospital, that we are rude and all that, so we try our best to discard all those misconceptions. Yeah. So, we give them respectful care from admission, from the first stage to the second stage, we continue to the third stage until they leave the ward to the post-natal ward.* Midwife 010.

#### Recognizing the autonomy of childbearing women

Some of the midwives mentioned how their participation in the training has helped them to involve childbearing women in caregiving decisions and recognized their views and rights to consent and to show empathy during care provision. Before the training, the midwives indicated that their approach to caregiving was expert-authority driven. They sometimes forcefully provided care to the women regardless of their dissents. After the training, the midwives mentioned that they involved the women in making decisions that are critical to their wellbeing during the caregiving process. The following quotes elucidate the forgoing point:

*… we are supposed to be checking the FH (fundal height) every fifteen (15) minutes, but because of pain, they won’t allow you. But after the training, you have to be by the patient’s side. If I put my hands there (during examination) and she says ‘No’, that I am in pain’ I have to understand …, manage the pain and leave her till when she is ready and decides to be examined, but I explain every procedure to them. But if it was at first, I would have forced the patient.* Midwife 003.

The trained midwives also report how they involve their client in their care:

*Now, we involve them [the women] in the care, and we ask them what they want. You know, sometimes when you are going to give infusion, you tell them we are going to cynto you (to give a Synticinom infusion). Ideally, you have to tell them what to expect when the infusion is on. That the contraction is going to increase and all that. So, you psych their mind before you set up the infusion. Sometimes the patient will tell you, ‘I don’t want the infusion’ but you just have to try to tell her maybe the importance of that drug that you are going to give her but if she insists that she doesn’t want it, it is her right. So right now, we are trying to involve them in the care that we are giving to them. We don’t want to impose on them and sometimes too, these days, when they get to the second stage, some people will try to just deliver on the floor, deliver at the first stage room. At first, we were insistent that you have to come to the second stage room, you have to do this, you have to lie like this but … after the workshop, we are respecting and accepting their views and choice. We try to make them feel comfortable.* Midwife 010.

### Health facility barriers to practising RMC

We asked the midwives to tell us the conditions acting as barriers to fully implementing RMC practices in their place of work. Their reports on the implementation challenges are organized under these two subthemes: policy and logistic constraint on alternative birthing positions and logistic constraints on ensuring privacy.

#### Policy and logistic constraints on alternative birthing positions

The midwives mentioned that the policy and the built environment in the hospital does not support the exploration of alternative birthing positions, and this has made it impossible for the midwives to assist the childbearing women to assume their preferred birthing position. The midwives were trained to help the women to deliver babies in the lithotomy position. Having participated in the RMC training program, they became aware of existing alternative birthing positions. They, however, indicated that the needed equipment to implement the alternative birthing positions are unavailable in their facility, making it impossible to put into practice the skills they acquired during the training. Below are some of their comments.

*The alternative birthing positions. It seems because we haven’t gotten the necessary equipment, all our patients have to take the lithotomy position. But when it comes to a patient wanting to step down and walk about a little, it’s been going on well, especially when a patient has no contra-indications of maybe, a rupture, or cord prolapse we allow the patient to walk around. Midwife 005.*

*… You know … here (referring to the facility), we have one position (chuckling), because we don’t have the necessary equipment for the other positions, so even if you try it … let’s say … squatting … we may have some issues. But as for bed delivery, we do that a lot because sometimes, when you are deciding whether to take the labouring woman elsewhere, you can’t because the head is [in the] vagina, so you have to do the delivery on the bed. Midwife 004.*

*We use the lithotomy position. The other positions...cannot be used on our wards. When we went for the training, they showed us the beds that they use and other things. And we realized that even if we want to do that we don’t have that kind of bed; the stirrups to support the knee. Midwife 011.*

#### Logistic constraints on ensuring privacy

Privacy during childbirth is important for childbearing women; therefore, during the training, the midwives were taught how to ensure privacy when childbearing women visit the hospital for delivery. During the follow-up interviews, the authors inquired about the childbirth privacy changes they implement. The midwives mentioned that they have had challenges because they lacked the logistics to ensure privacy for childbearing women in the open labour ward. They mentioned that the facility has only two screens, and it makes it difficult to provide intrapartum care for more than two women at a time. The following are some of the responses.

*The privacy screens [curtains] … for now, we have two (2) of them outside for patients so sometimes, if you have maybe three (3) labour cases, it means two will be provided with privacy and the others not. Midwife 002.*

*With the ward, providing privacy is one of our biggest challenges … we have fewer screens here. Our screens are not many. You may be using a screen for a patient, the other one too may need a screen but they both can’t have it at the same time so one has to be exposed whilst the other one is screened. So, if the screens are many, and I think each cubicle should have a screen so that the patient will have their privacy.* Midwife 009.

### Midwives’ recommendations for improving RMC practice

From the perspective of the midwives, we sought to understand what they consider to be critical recommendations to promote RMC practices in their place of work. They recommended that logistics for alternative birthing positions and privacy in the ward should be provided and all midwives and staff of the hospital should be taken through the RMC training program to encourage good practice. The voices of the midwives are captured under each of the subthemes as follows:

#### Logistics for alternative birthing positions

To enable them to implement the alternative birthing positions they were taught, the midwives requested that the labour ward at the facility should be equipped with the necessary supportive equipment.

*The recommendations I can make, or we need, are the positions kits required to enable us to implement the other positions we know. Because, just as we’ve been talking about, suppose we get these kits, we may not even revert to the lithotomy position anymore. As far as I am concerned, that is the most important challenge.* Midwife 005.

#### Logistics for privacy in the ward

The midwives had challenges ensuring privacy for childbearing women during childbirth. Therefore, they proposed that the hospital management should provide enough shower curtains. These are quotes from some of the midwives.

*The management should provide, maybe curtains like these (indicating) for all the patients, because when they are lying down, just walking around, others will not see their nakedness and all. I don’t know what they (the administration) are doing about it, but if they can provide those curtains, the shower curtain that we can just remove and wash it when there are some stains.* Midwife 010.

*They should provide us with the logistics, and if curtains and other things can be provided...so, now that we have a new facility and there are cubicles, they can provide curtains … for us to ensure a little privacy …* Midwife 012.

#### Training for all staff

The midwives mentioned that all midwives at the facility should be taught the RMC modules. Some mentioned that they, the initial beneficiaries of the training, should be allowed to organize workshops and share their knowledge with their colleagues. Additionally, they recommended that the training could target student-midwives in nursing training colleges.

*Yes, add other people to it. So, if maybe, I am suggesting maybe we the participants would be allowed to also, maybe organize workshops for the others and then get them on board.* Midwife 001.

*… The recommendation will be to get everyone [all staff] involved, so, at least, we will be able to share the ideas we had from the training, and then impart unto others … which will make healthcare delivery simple … Midwife 004.*

*The management should organize workshops for the whole department so that all our colleagues would be able to get the privilege to go to the workshop because it is very helpful. Midwife 010.*

*All staff from other facilities in the hospital too should have the training. And then, if it becomes very effective, which I know it will be because I have seen the effects, the positive effects myself, then maybe we can forward it to the training schools so that they are also be taught when they are in school. By the time they come out, they have what it takes to give the best of care to their patients. So that will be, and then on the hospital, I think the administration must also be involved. During the training, I didn’t see our director around. But I don’t know whether the next training she can be around or somebody, a representative of the administration, so that they know the importance of what we are learning. Then they can also come out with a policy or something to guide the whole program. It will help with the implementation.* Midwife 011.

## Discussion

We explored midwives’ experiences of applying RMC knowledge in their professional practice after participating in a four-day RMC training program.

Our study revealed that a refresher training course that seeks to inculcate in healthcare providers attitude, knowledge, and best practices of providing respectful maternity care to childbearing women have the potential for a positive impact. The data revealed that the training has improved the midwife-client relationship, made midwives better communicators on misconceptions that affect the caregiving practice and helped the midwives to recognize the autonomy of childbearing women. Having experienced the positive impact of the training on their practice, the midwives who participated in the training proposed that their colleagues should equally be trained and some suggested to become peer-teachers to their colleagues. Our results support a growing body of evidence that show that RMC short-course training programs can positively transform midwives to provide optimal maternity care in healthcare facilities without disrespecting and abusing childbearing women [9, 16, 17].

The findings revealed that, though the training has had a positive influence on maternity care practices of the midwives, conditions such as lack of hospital policy and inadequate support for alternative birthing positions and limited resources to ensure privacy for childbearing women act as barriers to the full application of RMC knowledge in the hospital. We have observed that the existing policy and infrastructure in the study setting and other healthcare facilities across the country support only the dorsal, supine, or lithotomy positions for non-instrumental vaginal deliveries. Although midwives have adequately been trained during the four-day workshop to assist childbearing women to deliver in alternative positions, they are faced with health system challenges they have no control over. This situation suggests that the training interventions need to be complemented with a review of existing policies in line with current maternity care practices and infrastructure enhancement to fully promote RMC in the hospital and other healthcare facilities with similar conditions in Ghana.

The study has the following strength and weaknesses. The midwives shared their views on their successes and challenges in implementing their new knowledge of RMC practices. These feedbacks are useful for the authors in planning for subsequent training and designing advocacy programs that address the identified policy and logistics challenges. The sample frame of the present study was limited by the number of midwives that participated in the training. To understand the impact of the training on midwifery practice on a large scale, we planned to collaborate with the management of the health facility to release more of the midwives to participate in subsequent training programs.

## Conclusion

The study aimed to explore midwives’ experiences of applying RMC knowledge in their professional practice after participating in a four-day RMC training program. Despite the report of some RMC implementation challenges, the midwives generally noted that the 4-day RMC training has had a positive impact on their maternity caregiving practice in the hospital. We, therefore, recommend that policies and programs aimed at addressing the issue of disrespect and abusive practices during maternity care should advocate and include the building of facilities that support alternative birthing positions and privacy of childbearing women during childbirth.

## Supplementary Information

**Additional file 1.** Interview Guide for Midwives.

## Data Availability

The interview transcripts used for the analyses in this study are available from the corresponding author on reasonable request.

## Abbreviations

RMC-M: Respectful Maternity Care-Module
RMC: Respectful Maternity Care
LMICs: Low and Middle-Income Countries
