# Supplementary material for: Midwives’ experiences of implementing respectful maternity care knowledge in daily maternity care practices after participating in a four-day RMC training
Source: BMC Nurs. 2021 Mar 10;20:39. doi: 10.1186/s12912-021-00559-6 (PMC7945050; doi:10.1186/s12912-021-00559-6)
Supplement: Supplementary file 1 — Additional file 1. Interview Guide for Midwives. [file 12912_2021_559_MOESM1_ESM.docx]

**INTERVIEW GUIDE (ENGLISH)**

Explain research to participant and seek consent.

**Biographical data**

1. Age (years):……………………………………………………….
2. Educational background:…………………………………..
3. Professional grade…………………………………………….
4. Religion:…………………………………………………………….
5. Marital status:……………………………………………………
6. Number of children:…………………………………………..
7. Years of working experience……………………………….
8. Units worked………………………………………………………

Thank you for sharing your background with me. Now, do I have your permission to start the recording?

**PUT ON YOUR RECORDER**

[If No, thank participant for time and end the session] [If yes, continue with the interview]

Now that the recording has started, please say “Yes” to confirm that you approve of me recording the interview

***Midwives interview guide***

- - - 1. How many deliveries have you conducted since you had the training on RMC-M
      2. Share with me your experiences with the provision of RMC

Probes

- In what ways have you been able to implement some of the strategies you learned about in your training?
- What are some of the strategies / procedures have you not been able to implement, and why?
- What challenges did you have providing respectful maternity care?
- In what ways did you provide dignified maternity care to mothers?
- What challenges did you have providing dignified maternity care?
  - - 1. How has been your communication with mothers after your training?
- In what ways has your communication with mothers improved?
- What challenges did you have communicating with mothers?
  - - 1. What positions do you use during first stage of labour?
- What positions do you use during second stage of labour?
  - - 1. In what ways have your care provision improved over the period?
      2. What are your recommendations for improvement?
      3. Is there any other thing you may like to share with me?

Thank you!
